# Impairment of photoreceptor ribbon synapses in a novel *Pomt1* conditional knockout mouse model of dystroglycanopathy

**DOI:** 10.1038/s41598-018-26855-x

**Published:** 2018-06-04

**Authors:** Marcos Rubio-Fernández, Mary Luz Uribe, Javier Vicente-Tejedor, Francisco Germain, Cristina Susín-Lara, Cristina Quereda, Lluis Montoliu, Pedro de la Villa, José Martín-Nieto, Jesús Cruces

**Affiliations:** 1 Departamento de Bioquímica, Instituto de Investigaciones Biomédicas “Alberto Sols” UAM-CSIC, Facultad de Medicina, Universidad Autónoma de Madrid, 28029 Madrid, Spain; 2 Departamento de Fisiología, Genética y Microbiología, Facultad de Ciencias, Universidad de Alicante, 03080 Alicante, Spain; 3 Departamento de Biología de Sistemas, Facultad de Medicina, Universidad de Alcalá, 28805 Madrid, Spain; 4 Departamento de Biología Molecular y Celular, Centro Nacional de Biotecnología, Consejo Superior de Investigaciones Científicas (CSIC), 28049 Madrid, Spain; 5 Centro de Investigación Biomédica en Red de Enfermedades Raras (CIBERER), Instituto de Salud Carlos III, 28029 Madrid, Spain; 6 Instituto Multidisciplinar para el Estudio del Medio “Ramón Margalef”, Universidad de Alicante, 03080 Alicante, Spain

## Abstract

Hypoglycosylation of α-dystroglycan (α-DG) resulting from deficiency of protein O-mannosyltransferase 1 (POMT1) may cause severe neuromuscular dystrophies with brain and eye anomalies, named dystroglycanopathies. The retinal involvement of these disorders motivated us to generate a conditional knockout (cKO) mouse experiencing a *Pomt1* intragenic deletion (exons 3–4) during the development of photoreceptors, mediated by the Cre recombinase expressed from the cone-rod homeobox (*Crx*) gene promoter. In this mouse, retinal α-DG was unglycosylated and incapable of binding laminin. Retinal POMT1 deficiency caused significant impairments in both electroretinographic recordings and optokinetic reflex in *Pomt1* cKO mice, and immunohistochemical analyses revealed the absence of β-DG and of the α-DG-interacting protein, pikachurin, in the outer plexiform layer (OPL). At the ultrastructural level, noticeable alterations were observed in the ribbon synapses established between photoreceptors and bipolar cells. Therefore, O-mannosylation of α-DG in the retina carried out by POMT1 is crucial for the establishment of proper synapses at the OPL and transmission of visual information from cones and rods to their postsynaptic neurons.

## Introduction

Dystroglycanopathies, recently renamed as muscular dystrophies-dystroglycanopathies (MDDGs), constitute a heterogeneous group of recessive congenital neuromuscular diseases. These disorders are associated with a wide clinical spectrum, and with different grades of severity of muscular impairment, brain malformation and ocular abnormalities^1^. The main underlying cause of these symptoms is a deficiency in the process of glycosylation (mainly O-mannosylation) of dystroglycan (DG), a key component of the dystrophin-glycoprotein complex (DGC) in muscular^2^ and central nervous system (CNS) tissues^3^. DG acts as a nexus between the extracellular matrix (ECM) and the actin cytoskeleton, and is composed of two subunits: α-DG and β-DG. α-DG is an extracellular protein anchored to the cell membrane by the β-DG transmembrane subunit^4^, while on the cytoplasmic side β-DG is anchored to the cytoskeletal protein dystrophin^5^. α-DG is a heavily glycosylated protein with the potential to bind, by virtue of its O-mannosyl glycans, to a large number of ECM proteins, including laminin and pikachurin^6^, the latter exclusive of the retina.

Protein O-mannosyltransferase 1 (POMT1), which is the closest phylogenetic relative of yeast Pmt4^7^, acts in a heteromeric complex with POMT2 to add in the endoplasmic reticulum the initial mannose unit^8^ of all O-mannosyl glycan cores that become covalently attached to α-DG, including laminin-binding residues^9^. Mutations in the *POMT1* gene may cause severe, type A1 MDDGs (MDDGA1; OMIM #236670), including Walker-Warburg syndrome (WWS)^10^ and muscle-eye-brain disease (MEB)^11^, both of them coursing with significant impairments in the muscle, nervous system and eye.

Visual dysfunctions and eye morphological alterations are typical features of the most severe MDDGs. A wide variety of ocular anomalies have been described in *POMT1*-related WWS and MEB patients including retinal malformations, vitreoretinal dysgenesis, optic nerve hypoplasia and blindness^1,12–15^. In-depth studies on the role of POMT1 in human visual function have not been undertaken, and the constitutive knockout (KO) *Pomt1* mutation results in embryonic lethality in the mouse^16^. Consequently, its function in the retina has remained elusive. Instead, the role of α-DG and its O-mannosylation in the visual system has been documented in different MDDG mouse models knocked out in different genes, such as those encoding DG^17^, glycosyltransferases POMGnT1^18,19^ and LARGE^20,21^, or the α-DG-interacting protein at the outer plexiform layer (OPL), pikachurin^22^.

In this work, we present the first report of a *Pomt1* conditional knockout (cKO) mutant mouse. Using this novel animal model, we focused our studies on the role of α-DG and its glycosylation in the structure and physiology of the retina. We show that the absence of POMT1-mediated glycosylation of α-DG in this tissue causes significant alterations of the ribbon synapses established between photoreceptors and their postsynaptic retinal neurons, at both structural and ultrastructural levels. We also demonstrate the relevance of this post-translational modification of α-DG for the correct transmission of visual information from the outer to the inner retina.

## Results

### Generation of a *Pomt1* retinal cKO mouse

**Figure 1:**
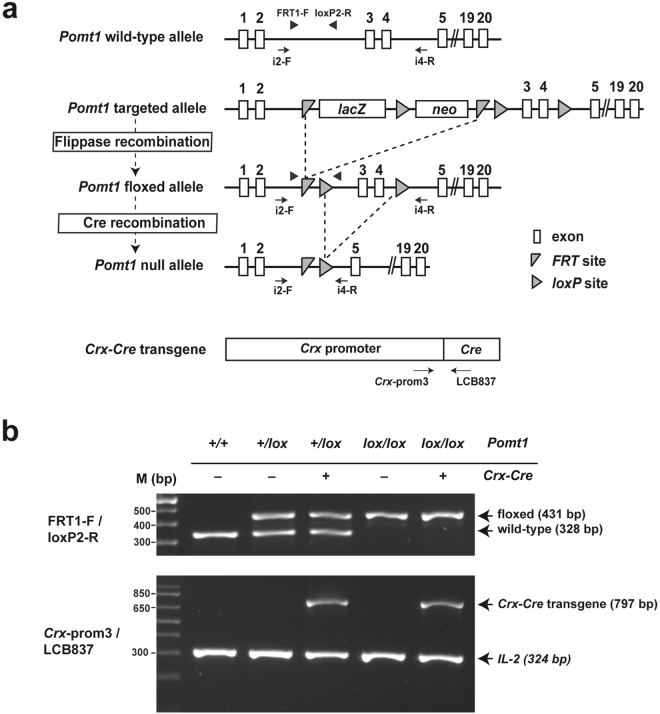
Generation of *Pomt1* retinal cKO mice. (**a**) Strategy for the generation of *Pomt1* null alleles. The *Pomt1* targeted allele was converted into a *Pomt1* floxed allele by flippase-mediated recombination removing the DNA segment flanked between FRT sites. In the resulting *Pomt1* floxed allele, exons 3 and 4 were flanked by *loxP* sites and were removed by Cre recombination to generate the *Pomt1* null allele (*Pomt1*^*−*^). Arrowheads indicate the position of primers to discriminate *Pomt1* wild-type and floxed alleles (used in **b**). Arrows show the annealing position of primers designed to assess Cre-mediated recombination in mouse retinas (used in Fig. 2a). The *Crx*-*Cre* gene fusion and the primers (arrows) used for its genotyping are represented at the bottom of the panel. (**b**) Results of PCR genotyping of *Pomt1* alleles (upper panel) and of the *Crx-Cre* gene fusion (lower panel). This analysis allowed the discrimination between *Pomt1* homozygous (wild-type or floxed) and heterozygous genotypes. The *Crx-Cre* genotyping yields a 797 bp band for the gene fusion and a 324 bp band for the control *IL-2* gene. Genotypes defined by the results are indicated above each lane. First lane, DNA ladder. Uncropped gel image is presented in Supplementary Fig. S6a.

To generate a retinal *Pomt1* cKO animal model, we designed a mating strategy based on the *Cre/loxP* system aimed at obtaining a mouse carrying homozygous *Pomt1* floxed alleles and the *Crx*-*Cre* transgene (*Pomt1*^*lox/lox*^/*Crx*-*Cre*^+^) (Fig. 1a and Supplementary Fig. S1). In this gene fusion, Cre recombinase activity is driven by the promoter of the cone-rod homeobox (*Crx*) gene, encoding a transcription factor essential for the development of photoreceptors whose expression becomes induced specifically in their precursors at embryonic day (E)12.5^23,24^. In this context, mating between floxed mice and a transgenic mouse line carrying the *Crx*-*Cre* gene fusion has allowed to obtain visually-impaired cKO mice ablated in particular genes specifically in photoreceptors, including those encoding the transcription factor Otx2^25^ and dystroglycan^17^, among many others. Following this strategy, a mouse bearing a *Pomt1* targeted gene and a flippase-expressing transgenic mouse were mated to obtain mice heterozygous for the *Pomt1*-floxed allele *(Pomt1*^+*/lox*^). These were interbred to generate homozygous floxed *Pomt1* (*Pomt1*^*lox/lox*^) animals and mice in parallel were crossed with *Crx*-*Cre*^+^ transgenic mice^25^ in order to obtain individuals heterozygous for the *Pomt1* floxed allele and the *Crx-Cre* transgene (*Pomt1*^+*/lox*^/*Crx*-*Cre*^+^). The mating between *Pomt1*^*lox/lox*^ and *Pomt1*^+*/lox*^/*Crx*-*Cre*^+^ mice finally generated *Pomt1*^*lox/lox*^/*Crx*-*Cre*^+^ mice (Supplementary Fig. S1). All these animals were subjected to genotyping by PCR analysis (Fig. 1b).

**Figure 2:**
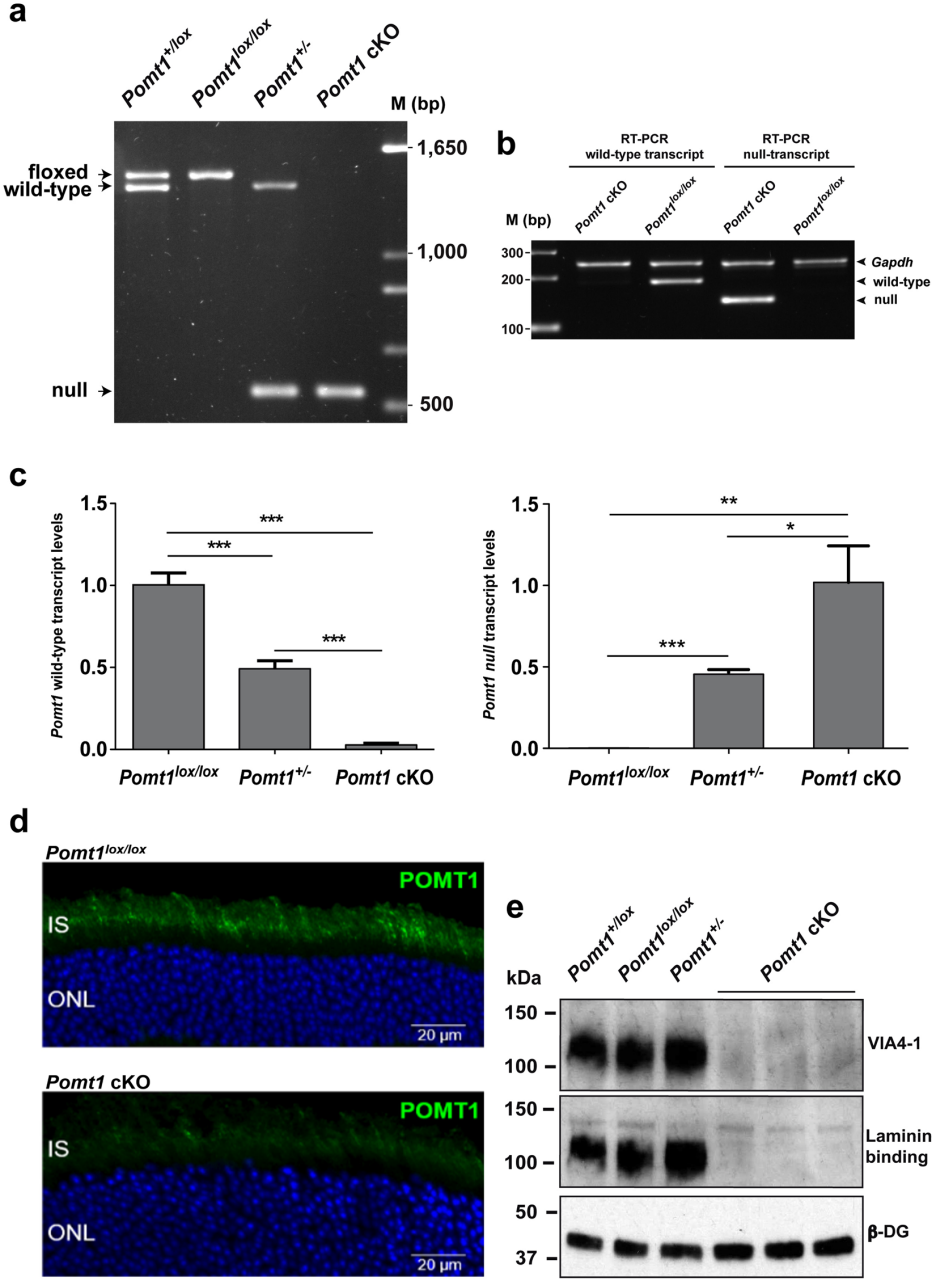
Analysis of *Pomt1* mRNA and protein expression, and α-dystroglycan glycosylation. (**a**) PCR analysis of *Pomt1* alleles in DNA extracted from mouse retinas: *Pomt1* floxed, 1,475 bp; *Pomt1* wild-type, 1,392 bp; *Pomt1* null, 545 bp. Genotypes defined by the results are indicated above each lane, abbreviated as indicated below. Last lane, DNA ladder. (**b**) Results of RT-PCR analysis designed to detect *Pomt1* transcripts. PCR products from the wild-type (182 bp) and null (136 bp) transcripts were detected in *Pomt1*^*lox/lox*^ and *Pomt1* cKO mice, respectively. *Gapdh* mRNA (238 bp) levels showed no differences across the different mouse retinas. (**c**) qRT-PCR analysis of RNA extracted from the retinas of *Pomt1*^*lox/lox*^, *Pomt1*^+/−^ and *Pomt1* cKO mice. The left graph shows the levels detected (mean ± SD, n = 4) of the *Pomt1* wild-type transcript, and the right graph the *Pomt1* null transcript levels (mean ± SD, n = 4). (Statistical significances: *p = 0.0299; **p = 0.0058; ***p < 0.001). (**d**) Expression of POMT1 in the mouse retina. Immunoreactivity of POMT1 in the inner segments of photoreceptors (green) of control mice (*Pomt1*^*lox/lox*^ in the picture) was virtually lost in the retina of *Pomt1* cKO mice. Nuclei were stained with DAPI (blue). Abbreviations: IS, inner segments; ONL, outer nuclear layer. (**e**) Western blotting analysis of α-DG glycosylation (VIA4-1, upper panel), laminin-binding ability (middle panel) and β-DG levels (lower panel) in WGA-enriched retinal extracts. Uncropped DNA gels and blot images are presented in Supplementary Figs S6a,b and S7. Genotype abbreviations: *Pomt1*^+*/lox*^ refers to the *Pomt1*^+*/lox*^*/Crx-Cre*^−^ genotype; *Pomt1*^*lox/lox*^ refers to *Pomt1*^*lox/lox*^*/Crx-Cre*^−^; *Pomt1*^+/−^ refers to *Pomt1*^+*/lox*^*/Crx-Cre*^+^; and *Pomt1* cKO refers to *Pomt1*^*lox/lox*^*/Crx-Cre*^+^.

In the retina, exons 3 and 4 of *Pomt1* were expected to be deleted in both alleles by Cre-promoted recombination, thus generating *Pomt1* null alleles. To test this, we performed PCR analysis on retinal DNA using a primer pair designed to detect all possible *Pomt1* alleles (Fig. 1a, arrows). This analysis revealed that only the *Pomt1* null allele was present in the retina of *Pomt1*^*lox/lox*^/*Crx*-*Cre*^+^ mice and, therefore, that retinal *Pomt1*^−/−^ conditional KO mice had been successfully achieved. By contrast, *Pomt1*^+*/lox*^/*Crx*-*Cre*^+^ littermates, used as controls, exhibited both *Pomt1* floxed and null alleles (hereafter named *Pomt1*^+/−^) in the retina, whereas animals lacking the *Crx-Cre* transgene (*Pomt1*^+*/lox*^ and *Pomt1*^*lox/*lox^) exhibited the original *Pomt1* wild-type and/or floxed alleles in this tissue (Fig. 2a).

At the mRNA level, transcription of the *Pomt1* null allele should result in a transcript lacking exons 3 and 4 and containing a premature stop codon in exon 5 (Supplementary Fig. S2). We performed RT-PCR and qRT-PCR assays using two different primer sets able to discriminate between *Pomt1* wild-type and null transcripts (Supplementary Fig. S2). Total RNA extracted from the retinas of *Pomt1* cKO, *Pomt1*^+/−^ and *Pomt1*^*lox/lox*^ mice allowed us to confirm that in *Pomt1* cKO mice only the *Pomt1* null transcript was present, and that *Pomt1*^*lox/lox*^ controls only expressed the wild-type transcript (Fig. 2b,c). In addition, heterozygous *Pomt1*^+/−^ mice were found to exhibit both wild-type and null transcripts in the retina (Fig. 2c). One-way ANOVA showed a significant effect of genotype on both *Pomt1* wild-type (F(2,9) = 365.036, p = 2.43·10^−9^) and null (F(2,9) = 61.905, p = 5.49·10^−6^) transcript levels. *Post hoc* test comparisons revealed significant differences among all genotypes (Fig. 2c). These data confirm that Cre-mediated *Pomt1* exon ablation results in the synthesis of *Pomt1* null transcripts in the retina of both *Pomt1* cKO and *Pomt1*^+/−^ mice. Additionally, immunostaining of POMT1 showed that, while this protein was located at the inner segments of photoreceptors of control mice, where the endoplasmic reticulum resides^26^, its immunoreactivity was virtually lost in the retina of cKO mice (Fig. 2d).

To study the effect of loss of POMT1 in retinal photoreceptors, we assessed the glycosylation status of α-DG by western blotting using the VIA4-1 antibody and laminin-binding assays, in wheat germ agglutinin (WGA)-enriched retinal extracts from *Pomt1* cKO mice and appropriate controls with different *Pomt1* genotypes. As shown (Fig. 2e), glycosylation of α-DG was lost together with its laminin-binding ability in *Pomt1* cKO mice as compared with control mice containing *Pomt1*^+^ and/or *Pomt1*^*lox*^ alleles. However, global levels of β-DG were unchanged in the retina of all animals studied, including *Pomt1* cKO mice (Fig. 2e). These results indicate that loss of POMT1 in the mouse retina drastically impairs α-DG glycosylation, and thereby laminin binding.

### Altered visual responses in *Pomt1* cKO mice

**Figure 3:**
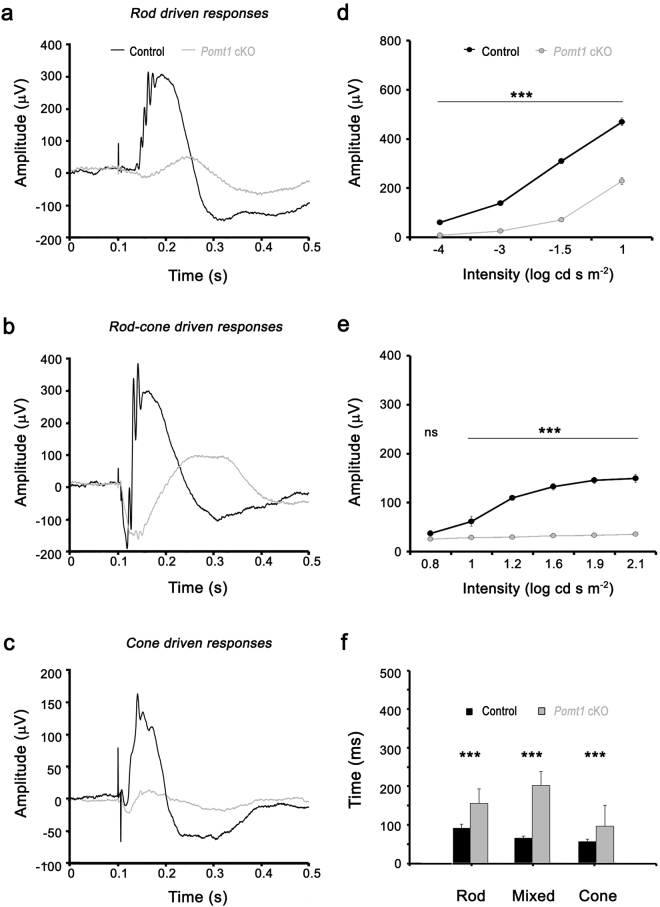
Functional retinal responses in *Pomt1* cKO mice. ERG responses recorded from representative control (black lines) and *Pomt1* cKO mice (gray lines) are shown (**a**–**c**). (**a**) Scotopic ERG rod response elicited by a −1.5 log cd∙s∙m^−2^ flash stimulus in control (*Pomt1*^+*/lox*^) and *Pomt1* cKO mice. (**b**) Mixed ERG rod plus cone responses showing the negative (a-wave) and positive (b-wave) deflections elicited by a 1 log cd∙s∙m^−2^ flash stimulus under scotopic conditions in control (*Pomt1*^+*/lox*^) and *Pomt1* cKO mice. (**c**) Photopic ERG cone responses elicited by a 1.6 log cd∙s∙m^−2^ flash stimulus in control (*Pomt1*^+*/lox*^) and *Pomt1* cKO mice. (**d**,**e**) Intensity-response curve analyses. The b-wave response amplitude (mean ± SEM, n = 18) is represented as a function of light intensity (log cd∙s∙m^−2^) from recordings obtained under scotopic (**d**) or photopic (**e**) conditions. Individuals analyzed included 6 *Pomt1*^*lox/lox*^, 6 *Pomt1*^+/−^ and 6 *Pomt1*^+*/lox*^ mice (control group), in comparison to 18 *Pomt1* cKO mice (mutant group). Response amplitudes showed statistical differences (***p < 0.001) (two-way ANOVA) between control and *Pomt1* cKO mice. (**f**) Implicit times of the b-wave averaged in rod, mixed and cone responses (mean ± SEM, n = 18; genotypes as in **d**,**e**) also showed statistical differences (***p < 0.001) between the two animal groups. Separate results obtained for each of the three control genotypes are presented in Supplementary Fig. S3.

To investigate the effect of POMT1 deficiency on visual function, we evaluated retinal physiology by recording electroretinographic (ERG) responses in controls (*Pomt1*^+/−^, *Pomt1*^*lox/lox*^ and *Pomt1*^+*/lox*^ mice) and in *Pomt1* cKO mice. No differences were observed between the three control genotypes, as shown in Supplementary Fig. S3. The scotopic and photopic ERG responses elicited by different stimulus intensities in experimental animals are shown in Fig. 3. The ERG responses to light intensities of −1.5 (Fig. 3a) and 1 log cd·s·m^−2^ (Fig. 3b) recorded in dark-adapted *Pomt1* cKO mice were severely reduced when compared with those of control mice. Average data on ERG b-wave amplitudes showed significant differences between control and *Pomt1* cKO mice (Fig. 3d) (F(1,3) = 965.8, p = 0.00004, two-way ANOVA, Bonferroni posttests, n = 18 animals per group). Notably, we further observed a significant delay in the scotopic and mesopic b-waves, as reflected by their longer implicit time (Fig. 3f; “Rod” and “Mixed”, respectively) (p = 0.0002 (rod) and 0.0018 (mixed), unpaired t-test, Welch’s corrected t-test). However, the a-wave amplitudes were quite similar between *Pomt1* cKO and control mice (Fig. 3b, negative deflection), thus indicating a normal function of photoreceptors. Altogether, these results suggest an impairment in visual signal transmission from rod photoreceptors to rod bipolar cells. We also assessed the ERG responses of control and *Pomt1* cKO mice under photopic conditions (Fig. 3c,e). The ERG b-wave elicited in light-adapted control mice was higher than that in *Pomt1* cKO mice (Fig. 3c), in the sense that a significant decrease in the b*-*wave amplitude was observed in *Pomt1* cKO mice for any tested light intensity under photopic conditions (Fig. 3e) (F(1,5) = 612.3, p = 0.00004, two-way ANOVA, Bonferroni posttests, n = 18 animals per group), together with a significant increase in the photopic b-wave implicit time (Fig. 3f; “Cone”) (p < 0.0001, Student’s t-test, Welch correction). These results reflect an abnormal signal transmission from cone photoreceptors to cone bipolar cells in *Pomt1* cKO mice.

**Figure 4:**
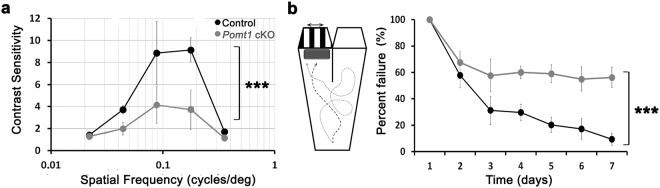
Visual responses in *Pomt1* cKO mice. (**a**) Optokinetic responses of control and *Pomt1* cKO mice. Contrast sensitivity is represented as a function of spatial frequency as the mean ± SD (n = 7). The control group included 3 *Pomt1*^+*/lox*^, 1 *Pomt1*^*lox/lox*^ and 3 *Pomt1*^+/−^ mice, and the mutant group included 7 *Pomt1* cKO mice. Statistical differences (***p < 0.001, two-way ANOVA) were found for intermediate spatial frequencies: 0.044, 0.088, and 0.177 cycles/degree. (**b**) Behavioral visual responses obtained in the water-maze test are represented. Left: drawing of the water-maze device where the discontinuous line depicts a representative correct response and the continuous line a representative failure response. Right: percent of failure responses (mean ± SEM, n = 7; genotypes as above) shown as a function of the testing day. Statistically significant differences (***p < 0.001) were observed from the 4^th^ day of testing.

We also evaluated the optokinetic reflex in control and *Pomt1* cKO mice. Responses induced by screen rotation at different spatial frequencies of alternating white and black stripes were significantly lower in *Pomt1* cKO mice than in control animals (Fig. 4a). Contrast sensitivity in the range 0.044–0.177 cycles/degree was weaker (F(1,2) = 13.04, p = 0.0009, two-way ANOVA, Bonferroni posttests, n = 7 animals per group) in *Pomt1* cKO mice than in control mice, which indicated a strong visual impairment in these animals. Finally, visual performance was also assessed by the water-maze test in both groups. While control animals improved their success rate along the testing days, *Pomt1* cKO mice failed to learn due to their visual impairment (Fig. 4b). In control animals, the average percentage of failure in reaching the platform located below the visual stimulus decreased from the 2^nd^ day of testing, reaching 10% failures by the 7^th^ day of experimental testing. By contrast, although the percentage of failures also decreased in *Pomt1* cKO mice, these were unable to reach the platform in 50% of trials by day 7^th^ after initiating the test. Significant differences between both animal groups (F(1,3) = 51.16, p = 0.00004, two-way ANOVA, Bonferroni posttests, n = 7 animals per group) were observed from the 4^th^ day after the experiment was started. These results reflect a remarkable impairment of visual performance in *Pomt1* cKO mice.

### Structural changes in the outer retina of *Pomt1* cKO mice

**Figure 5:**
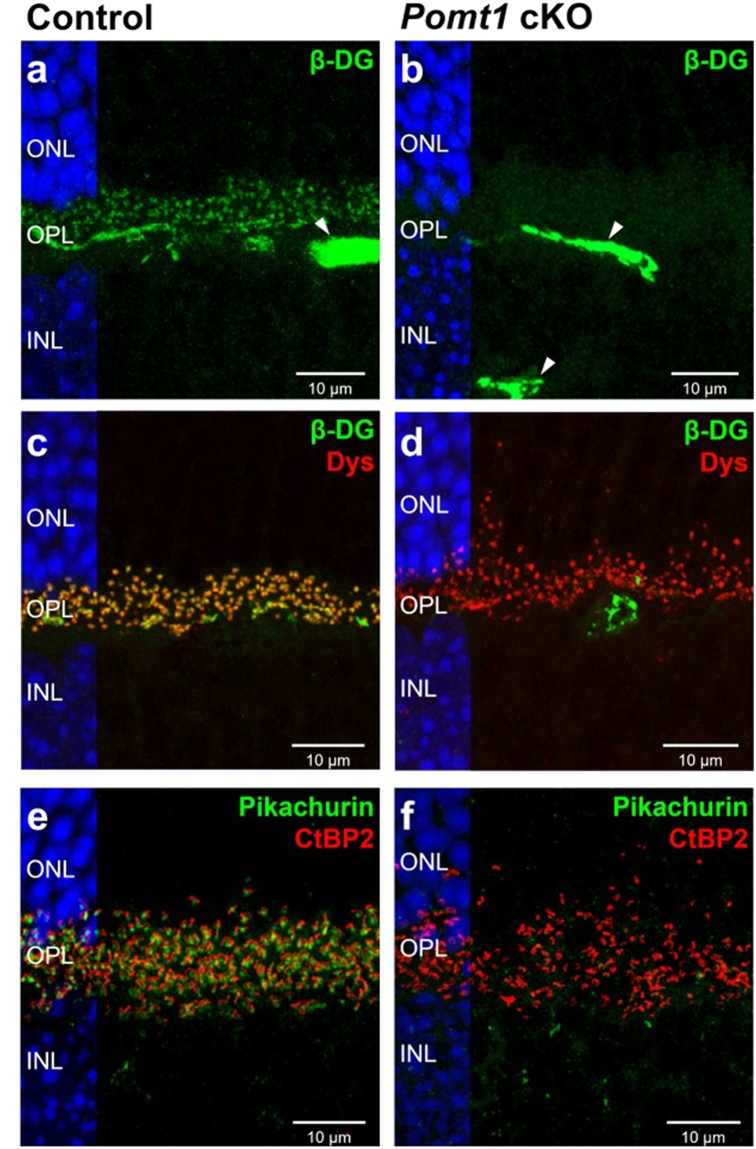
Expression and localization of presynaptic-terminal and associated proteins in the OPL of *Pomt1* cKO mice. Retinal sections of control (*Pomt1*^+*/lox*^) (**a**,**c**,**e**) and *Pomt1* cKO (**b**,**d**,**f**) mice were single or double immunostained with antibodies against β-dystroglycan (β-DG; **a**–**d**; green), dystrophin (Dys; **c**,**d**; red), pikachurin (**e**,**f**; green) and CtBP2 (**e**,**f**; red). The nuclei of all sections were stained with DAPI, and are shown in blue on the left side of each micrograph. β-DG was absent in the OPL of *Pomt1* cKO mice (**b**,**d**), although its expression was unchanged in retinal blood vessels as compared with control animals (**a**,**b**; arrowheads). Abbreviations: ONL, outer nuclear layer; OPL, outer plexiform layer; INL, inner nuclear layer.

Next, we examined the retinal morphology of control and *Pomt1* cKO mice. No obvious morphological differences in cell layer organization could be observed in retinal sections of *Pomt1* cKO animals stained with hematoxylin plus eosin (HE), or with DAPI. However, the thickness of the outer nuclear layer (ONL) and the OPL was significantly less in *Pomt1* cKO mice than in controls (Supplementary Fig. S4). To evaluate the effect of conditional knockout of *Pomt1* on the structure of the OPL, we performed confocal microscopy studies using antibodies against constituent elements of the DGC and associated proteins. In the retina of control mice, β-DG residing in the presynaptic membrane of photoreceptors displayed a punctate staining pattern along the OPL (Fig. 5a), as expected, whereas in the retina of *Pomt1* cKO mice β-DG immunoreactive dots were completely absent in this layer (Fig. 5b). Dystrophin was found along the OPL in both control and cKO animals, colocalizing with β-DG in the retina of control animals (Fig. 5c), but with a certain misalignment of immunoreactive dots in the retina of *Pomt1* cKO mice (Fig. 5d) with respect to the normal arrangement of dystrophin puncta in control mice (Fig. 5c). Immunostaining of pikachurin yielded a pattern of immunoreactive dots lying adjacent to synaptic ribbons stained with antibodies to CtBP2 in the OPL of control mice (Fig. 5e) but, by contrast to dystrophin, pikachurin immunoreactivity was completely absent in *Pomt1* cKO animals (Fig. 5f). Additionally, an intense fluorescence signal from β-DG and dystrophin was found in retinal blood vessels and the inner limiting membrane that was unchanged in the *Pomt1* cKO retina (Supplementary Fig. S5). These results indicate that loss of POMT1 in photoreceptors does not affect the structure of the innermost layers of the mouse retina.

**Figure 6:**
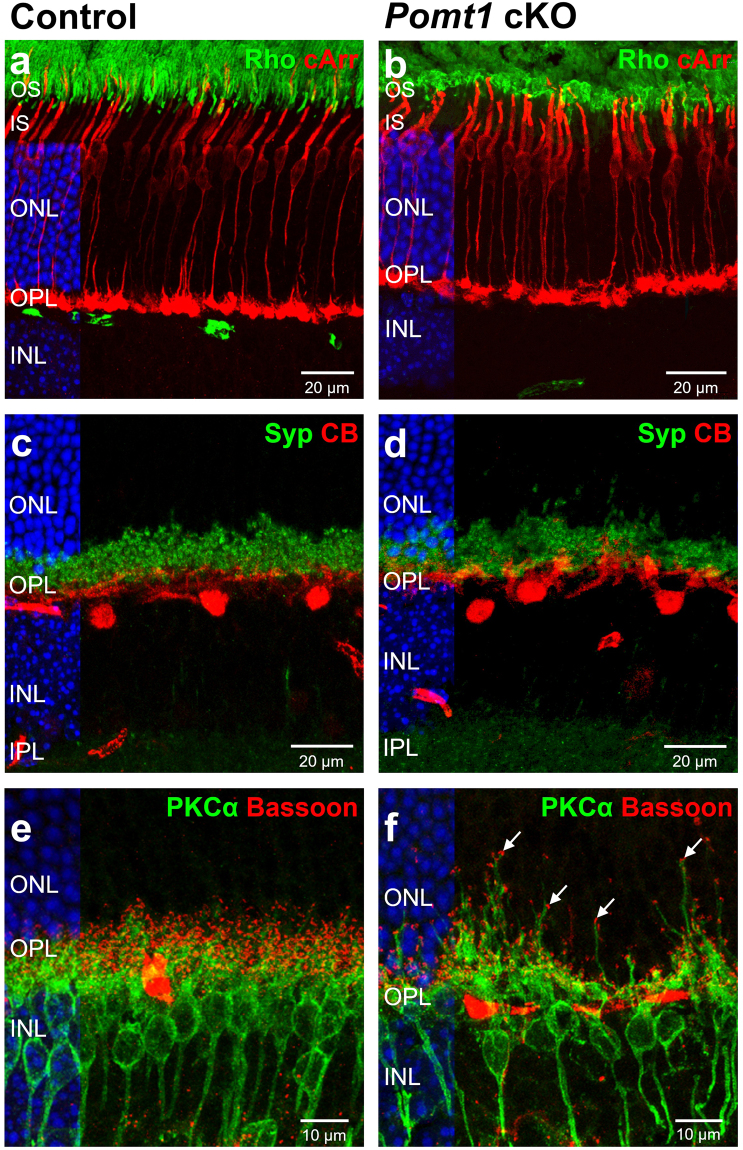
Structural alterations in outer retinal neurons of *Pomt1* cKO mice. Immunohistochemical analysis was carried out using antibodies against rhodopsin (Rho; green), which labels the outer segments of rods, and cone arrestin (cArr; red), a specific marker for cones (**a**,**b**). The distribution pattern of synaptophysin (Syp; green), a presynaptic protein located in the axon terminals of photoreceptors, and calbindin (CB; red), which labels horizontal cells (**c**,**d**), are also shown. Synaptic contacts between presynaptic terminals were labeled with antibodies to bassoon (red), and dendrites of rod bipolar cells with antibodies to protein kinase Cα (PKCα; green) in the retinas of control (*Pomt1*^+*/lox*^) and *Pomt1* cKO mice (**e**,**f**). Bipolar cell dendrite sprouting into the ONL was found in certain areas of the retina of mutant animals (**f;** arrows). Nuclei stained with DAPI are shown in blue on the left side of each micrograph. Abbreviations: OS, outer segment; IS, inner segment; ONL, outer nuclear layer; OPL, outer plexiform layer; INL, inner nuclear layer; IPL, inner plexiform layer.

**Figure 7:**
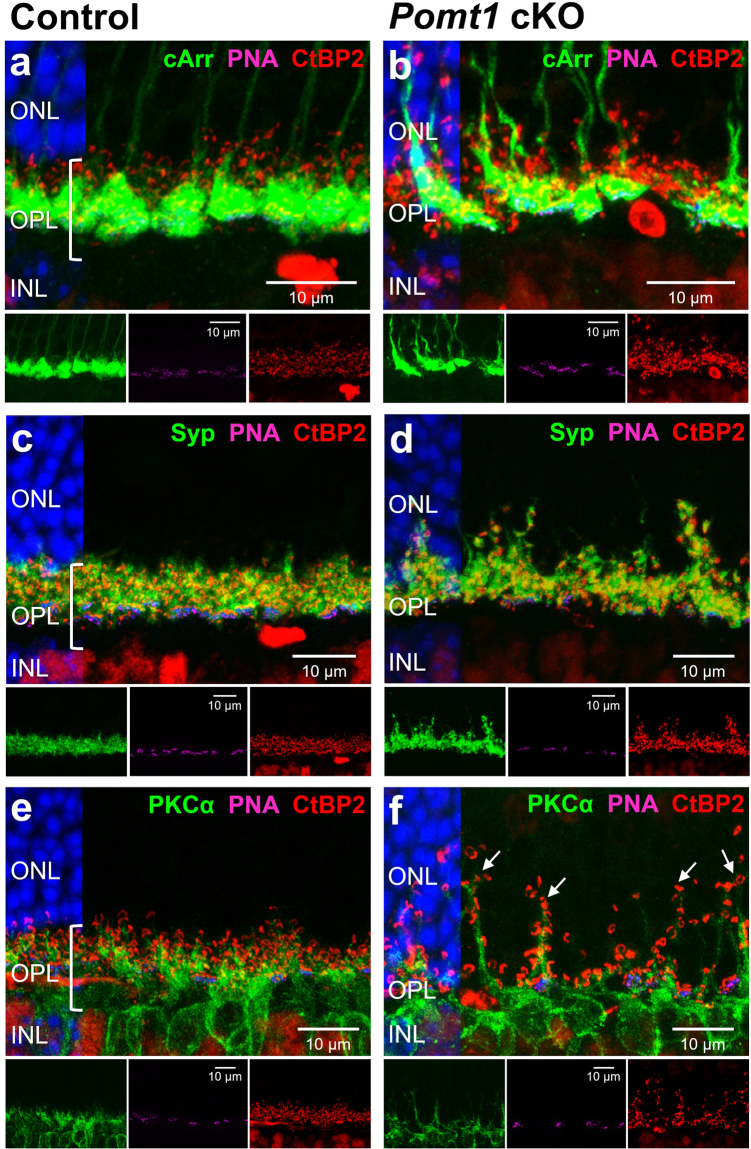
Presynaptic and postsynaptic terminals in the OPL of control and *Pomt1* cKO mice. Retinal sections from control (*Pomt1*^+*/lox*^) (**a**,**c**,**e**) and *Pomt1* cKO (**b**,**d**,**f**) mice were stained with antibodies against CtBP2 (red), which labels synaptic ribbons of cones and rods, and with peanut agglutinin (PNA; magenta), a lectin that labels the base of cone pedicles. Additionally, they were immunostained for cone arrestin (cArr; **a**,**b**; green), a cone-specific presynaptic protein, synaptophysin (Syp; **c**,**d**; green), a presynaptic protein labeling cone pedicles and rod spherules, and protein kinase Cα (PKCα; **e**,**f**; green), which labels rod bipolar cells. The nuclei of all sections stained with DAPI are shown on the left side of each micrograph. In the retina of *Pomt1* cKO mice, loss of normal alignment of synaptic proteins in the outer half of the OPL was observed for CtBP2 (**b**,**d**,**f**), Syp (**d**) and PNA (**b**,**d**,**f**) labeling (see insets), as compared with the OPL of control mice (**a**,**c**,**e**; bracket and insets). Besides, considerable sprouting of bipolar cell dendrites protruding into the ONL was revealed by PKCα immunostaining in *Pomt1* cKO mice (**f**; arrows) as compared with control mice (**e**). Abbreviations: ONL, outer nuclear layer; OPL, outer plexiform layer; INL, inner nuclear layer.

We used antibodies against rhodopsin and cone arrestin to further examine the morphology of cells involved in the first synapse of the visual pathway. These photosensitive proteins were properly distributed in the outer segments of rods and cones, respectively, although some retraction of these structures in rods and a shortening of cone axons were observed in *Pomt1* cKO mice when compared with controls (Fig. 6a,b). To investigate possible alterations in the structure of synapses established between photoreceptor axon terminals and their postsynaptic (horizontal and bipolar) cells, we performed double immunostaining using antibodies against the presynaptic proteins synaptophysin and bassoon, and against calbindin (a horizontal cell marker) and protein kinase C (α isoform, a rod bipolar cell marker). No obvious differences were observed between the retinas of control and *Pomt1* cKO mice in the punctate immunostaining pattern of synaptophysin or in the morphology and number of horizontal cells (Fig. 6c,d). However, although we did not find differences in the association between the presynaptic marker bassoon (Fig. 6e,f) or the postsynaptic marker mGluR6 (data not shown) and dendrites of rod bipolar cells, a misalignment of presynaptic terminals of photoreceptors in the OPL was observed in specific areas of the retina in *Pomt1* cKO mice, which was associated with an abnormal sprouting of dendrites from bipolar cells protruding into the ONL (Figs 6f and 7f; arrows). These observations were further corroborated by fluorescent labeling for the synaptic-ribbon marker CtBP2, the presynaptic-vesicle protein synaptophysin, and peanut agglutinin (PNA), which labels the base of cone pedicles (Fig. 7). As shown, in the retina of *Pomt1* cKO mice a loss of the normal arrangement of synaptic proteins in the outer half of the OPL was observed upon CtBP2 (Fig. 7b,d,f) and Syp (Fig. 7d) immunostaining, as compared with the OPL of control mice (Fig. 7a,c,e). Also, the normally discontinuous PNA labeling in this layer in control mice (Fig. 7a,c,e) showed a conspicuous misalignment in *Pomt1* cKO mice (Fig. 7b,d,f).

**Figure 8:**
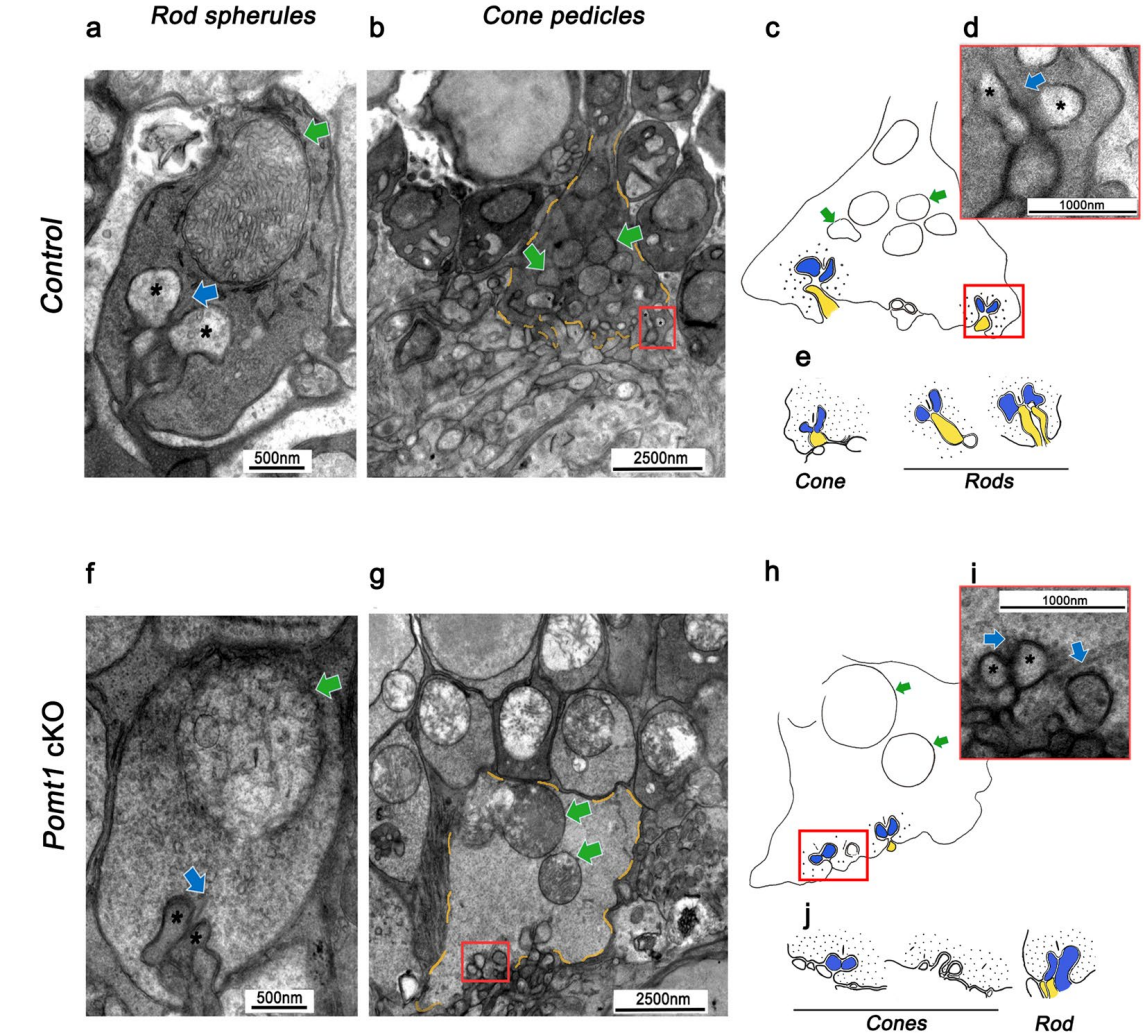
Electron microscopy images of photoreceptor axon terminals in *Pomt1* cKO mice. Rod spherules and cone pedicles are shown from control (*Pomt1*^*lox/lox*^) (**a**–**e**) and *Pomt1* cKO (**f**–**j**) mice. At the rod spherule of control mice (**a**), processes from horizontal cells (asterisks) were located laterally apposed to the synaptic ribbon (blue arrow), and dendritic processes from rod bipolar cells were located below the ribbon. By contrast, horizontal cell processes in *Pomt1* cKO mice (**f**) failed to reach their proper position relative to the rod synaptic ribbon, and dendritic processes from rod bipolar cells were barely visible at the synaptic cleft. At the cone pedicle (yellow dashed line in **b** and **g**; schematized in **c** and **h**, respectively), the number of invaginations (synaptic complexes) was higher in control (**b**) than in *Pomt1* cKO (**g**) mice, and mitochondria (green arrows) were more abundant near the synaptic complexes of control (**b**,**c**) than of *Pomt1* cKO (**g**,**h**) mice. Details of the synaptic complex at cone pedicles (red boxes in **b** and **g**) are schematized in panels c and h, and shown at a higher magnification in **d** and **i** for control and *Pomt1* cKO mice, respectively. As noticeable, the lateral processes from horizontal cell (asterisks) and basal process from bipolar cells exhibited a normal appearance in control mice (**c**), but seemed disorganized relative to the synaptic ribbon (blue arrows) in *Pomt1* cKO mice (**i**). Drawings in **e** and **j** schematize the differences observed between cone and rod synaptic triads of control and *Pomt1* cKO mice, respectively.

Synaptic alterations at the OPL level were further examined by electron microscopy. At a first glance, the structure of synaptic complexes in the conditional mutant mice appeared abnormal (Fig. 8). In both control and *Pomt1* cKO animals, presynaptic terminals exhibited a bar-like synaptic ribbon, but dendritic projections from bipolar and horizontal cells appeared to be altered in *Pomt1* cKO mice. In control animals, synaptic structures in both rod and cone axon terminals showed a clearly normal shape, with horizontal cell processes located laterally opposed to the synaptic ribbon, and with bipolar cell processes pointing to the base of the ribbon (Fig. 8a–e). By contrast, in *Pomt1* cKO mice, horizontal cell dendritic projections barely reached their location laterally to the synaptic ribbon, and bipolar cell dendritic projections failed to meet photoreceptor synaptic terminals (Fig. 8f–j). The synaptic complex was absent in those rod spherules lying most distant from the OPL. Concurrent with these alterations, mitochondria in both rod and cone axon terminals in *Pomt1* cKO mice exhibited evident alterations, appearing swollen and showing a severe disruption of their cristae (see for comparison Fig. 8a,f). Also, the number of mitochondria was reduced in *Pomt1* cKO mice and their distance from the synaptic complex was increased in cone pedicles (see for comparison Fig. 8b,g). Overall, these results reflect an abnormal organization of synapses between rod and cone axon terminals and their postsynaptic cells in our *Pomt1* cKO mice.

## Discussion

MDDGs are rare neuromuscular congenital dystrophies with a total of 18 associated genes identified so far, all of them related to the O-mannosylation pathway of the DG α subunit^2,27^. This modification is essential for linkage via α-DG of cytoskeletal components to the ECM in muscle and brain cells, and crucial for proper synapse formation and function in the retina^27–29^. Among those genes, *POMT1* encodes the first enzyme acting on the α-DG O-mannosylation process, and it has been shown that mutations in this gene may cause severe MDDGs, including WWS and MEB, usually coursing with serious ocular abnormalities^13,15,30,31^. The *Pomt1* gene has been shown by our group to be expressed at the mRNA level during mouse embryogenesis in the nervous system, muscle and eye^16,32^, coincidently with tissues mostly affected in patients with such diseases. As well, we have recently reported expression of *Pomt1* at both mRNA and protein levels in the adult retina of the mouse and a variety of mammalian species^33^. In this context, we have proposed that POMT1 deficiency is related to ocular abnormalities detected in patients with these diseases^32^, an issue whose addressing led us to generate the *Pomt1* cKO model reported in the present work.

Targeted disruption of MDDG-related genes like *DG*^34^, *POMT1*^16^, fukutin^35^ or FKRP^36^ is lethal during early mouse embryogenesis, and is consistent with a properly glycosylated, and hence functional, α-DG being essential for the formation and integrity of Reichert’s membrane^34^. To overcome this problem, we generated homozygous *Pomt1*-floxed mice, which were crossed with a transgenic mouse line carrying a *Crx-Cre* gene fusion. This transgenic line has been used to successfully generate photoreceptor-specific cKO mice in a wide variety of genes^17,25,37–41^. As a result, we obtained animals harboring a *Pomt1* intragenic deletion in retinal tissue (conditional *Pomt1*^−/−^ genotype, or *Pomt1* cKO), which at the mRNA level caused the appearance of a *Pomt1* null transcript containing a premature stop codon. In the *Pomt1* cKO retina, α-DG glycosylation was virtually lost together with its ability to bind laminin. Unfortunately, for lack of an appropriate antibody working in immunohistochemistry against α-DG (either glycosylated or core α-DG), we could not verify this result at the structural level on mouse retinal sections. By contrast, both α-DG properties remained in heterozygous *Pomt1*^+/−^ mice, consistent with the recessive character of *POMT1*-associated MDDGs. Our *Pomt1* cKO mice were thus genotypically and biochemically as expected, and POMT1 activity was shown to be crucial for interaction of retinal α-DG with laminin, as previously demonstrated in muscle and brain^42^. Furthermore, we failed to detect alterations in the innermost layers of the retina, or in vital organs of mice, demonstrating that the *Pomt1* gene had been specifically knocked out in retinal photoreceptors.

*Pomt1* cKO mice display a normal a-wave, but show a clear reduction in amplitude and a longer implicit time of the b-wave in ERGs recorded under scotopic and photopic conditions. These results reflect an impairment of visual transmission from rods and cones to their postsynaptic bipolar cells, originating in the OPL at the photoreceptors presynaptic side. This ERG dysfunction is comparable to that caused by loss of DG in the OPL^17^, but is more severe than that found in *Pomgnt1* KO^18,19^ and *Large*^*myd*^ spontaneous KO mice^20,21^ and *Pikachurin* null mice^22^ in their ERG scotopic b-wave responses. The higher severity of POMT1 loss could be attributable to the importance of the first limiting step in α-DG O-mannosylation carried out (as a heterodimer) by POMT1 and POMT2 enzymes^8^. Indeed, these are the only two proteins simultaneously involved in the synthesis of the three main O-mannosylglycan structures of α-DG, designated as M1, M2 and M3 cores^9^.

Until now, no data were available on the effect of DG absence on the optokinetic reflex or visual behavior, both of which were impaired in *Pomt1* cKO mice, and to a greater extent in *Pikachurin* KO mice^22^. The latter did not show noticeable alterations with relatively large angle stripes, but their sensitivity to small angle stripes was significantly impaired^22,43^. The decrease in information reaching retinal bipolar cells from photoreceptors may explain our results. Additionally, failure of learning by *Pomt1* cKO mice in the water-maze test could be attributed to a remarkably impaired visual performance.

In similarity to *DG* cKO^17^ and *Pikachurin* KO^22^ mice, functional deficiencies in *Pomt1* cKO mice were not accompanied by obvious malformations of retinal layers. This is in contrast to the loss of retinal laminar structure, thinner retina, retinal detachment, and decreased density of photoreceptors and ganglion cells observed in MDDG mouse models deficient in POMGnT1^18^, fukutin^35^ or LARGE^21^. We evaluated potential alterations in DGC components at the OPL level in *Pomt1* cKO mice by immunohistochemistry. β-DG and pikachurin, the main α-DG-interacting ECM protein, were completely absent at the OPL in *Pomt1* cKO mice, although the intracellular component dystrophin was unaltered. The loss of β-DG found at the OPL contrasts with the result that global retinal levels of β-DG were not detectably decreased by western blotting, although this could be accounted for, at least in part, by its immunoreactivity being unaltered in the ILM and retinal blood vessels, where its expression appears predominant. Nevertheless, the absence or presence of β-DG in the OPL of MDDG animal models is controversial, and in this light our results are similar to those obtained in *Pikachurin* KO and *Large*^*vls*^ mice^17,21^, but are at variance with other null models, such as the *Pomgnt1* KO and the *Large*^*myd*^ and *Large*^*PB*^ mice, in which β-DG was retained in this retinal layer^44–46^. Recently, it has been suggested that proper O-mannosylation of α-DG could be necessary for its proper folding, and that the lack of this post-translational modification would trigger its degradation in the endoplasmic reticulum^47^. Regarding pikachurin, its absence in the OPL of *Pomt1* cKO mice is in concordance with results obtained in *DG* cKO, *Pomgnt1* KO and *Large*^*myd*^ mice^17,45,48^. Since its encoding gene is normally expressed at the mRNA level in the retina of *Pomgnt1* KO and *Large*^*myd*^ mice, but pikachurin protein is not detected by western blotting^45^, the latter is expected to have as well become degraded, upon failure of its interaction with α-DG. Taken altogether, our data strongly suggest that the first step in O-mannosylation of α-DG carried out by POMT1 is crucial for both the correct accumulation of pikachurin in the OPL and the proper localization of β-DG at the presynaptic terminals of photoreceptor cells.

A misalignment and retraction of presynaptic terminals of photoreceptors and dendritic sprouting of bipolar cell into the ONL were observed in immunolabeled retinal sections of *Pomt1* cKO mice. These abnormalities were confirmed at the ultrastructural level by electron microscopy, where it was observed that dendritic processes of bipolar and horizontal cells were not inserted into the invaginations of ribbon synapses, resulting in a gap between the pre- and post-synaptic active zones. These defects have been previously associated with a delayed and reduced b-wave amplitude in the ERG of mutant mice impaired in different components of the DGC^49^, as it was the case for our *Pomt1* cKO mice.

Electron microscopy analysis showed that mitochondria in rod and cone synaptic terminals appeared reduced in number and swollen, with severe disruption of their cristae. Mitochondria in the OPL are also swollen in *Large*^*myd*^ mice, which additionally show numerous, disrupted cristae in *Large*^*vls*^ mutants^21^, as occurs in other rodent models of retinal degeneration^50–52^. Impaired electron transport and oxidative stress^53^ at the presynaptic terminals could consequently occur in our *Pomt1* cKO mutants, as proposed for the *rd10* mouse model of retinitis pigmentosa^54^.

In summary, lack of the first enzyme acting on α-DG glycosylation, POMT1, prevents the correct formation of synaptic complexes at the OPL, as evidenced in this work at structural and ultrastructural levels. Consequently, transmission of visual signals from rods and cones to their postsynaptic bipolar cells is severely impaired as a likely consequence of the loss of glycosylation-dependent interaction between α-DG and ECM proteins, such as pikachurin and laminin. Our present findings indicate that POMT1 is a key O-mannosyltransferase relevant for the formation and function of synapses between photoreceptors and their postsynaptic bipolar cells, its mutational loss thereby mimicking a good part of the retinal pathology found in WWS and other *POMT1*-associated MDDGs.

## Materials and Methods

### Animal care

All procedures were performed according to the Spanish and European legislation, and experiments were approved by the Ethics Committees of the Universidad Autónoma de Madrid and Universidad de Alcalá. Experiments were carried out in mice ranging from 3 to 5 months of age.

### Generation of *Pomt1* cKO mice

The mating strategy used with this purpose is schematized in Supplementary Fig. S1. *Pomt1*-targeted mouse embryonic stem cells were obtained from the EUCOMM program (http://www.mousephenotype.org/) and were aggregated with morulas (E2.5) from CD1 mice to generate chimeric animals. These were mated with wild-type mice and their offspring were genotyped for transmission of the *Pomt1* targeted allele by PCR amplification of the *lacZ* sequence using primers lacZ-F and lacZ-R. To generate the *Pomt1* floxed mouse line, transgenic mice bearing the flippase recombinase transgene (*FLP*) were crossed with mice carrying the *Pomt1* targeted allele. Mice with successful flippase-mediated recombination were selected by PCR using primers FRT1-F and loxP2-R. This PCR reaction was used subsequently to discriminate between the different *Pomt1* genotypes. Transgenic *Crx-Cre*^+^ mice^25^ were purchased from the RIKEN BioResource Center (http://mus.brc.riken.jp/en/). For genotyping of mice carrying the *Crx-Cre* transgene, we used the primers recommended by RIKEN, Crx-prom3 and LCB837 (Cre2). Also, we used the control primers oIMR0042 and oIMR0043 to amplify an interleukin-2 (*IL2*) gene fragment. For the assessment of intragenic *Pomt1* deletion in mouse retinal genomic DNA, retinas were extracted from the eyes of 5-month-old mice. DNA isolation was performed following the phenol/chloroform-free method described previously^55^. Primers Pomt1-i2-F and Pomt1-i4-R were used to distinguish between *Pomt1* wild-type, floxed and null alleles. All primer sequences and diagnostic PCR products are listed in Supplementary Table S1.

### RT-PCR and qRT-PCR analysis of *Pomt1* transcript variants

Retinas were extracted in the presence of RNA*later* (Ambion, Austin, TX, USA). Total RNA was obtained using the RNeasy Mini kit (Qiagen, Valencia, CA, USA), and reverse transcription of 150–200 ng of RNA was carried out using the qScript cDNA kit (Quanta Biosciences, Gaithersberg, MD, USA). For RT-PCR, we used two different primer sets to distinguish between the *Pomt1* wild-type (E2/3-F and E4/5-R primers) and null (E2/5-F and E5-R primers) mRNA transcripts. For each PCR reaction, the corresponding *Pomt1* primer pair was used together with control primers Gapdh-F and Gapdh-R.

For qRT-PCR, cDNA was obtained using the High Capacity cDNA Reverse Transcription Kit (Applied Biosystems, Foster City, CA, USA). Quantitative PCR was performed in a 7900 Fast Real-Time PCR System (Applied Biosystems) using the above *Pomt1* primers and SYBR Green PCR Master Mix. The *Actb* gene was used as an endogenous housekeeping gene for normalization (primers Actb-F and Actb-R). Statistical analyses were performed using the SPSS software package (IBM Corp., v21.0). Differences in *Pomt1* transcript levels between different genotypes were tested using one-way ANOVA. Both expression data were normally distributed, but the *Pomt1* wild-type transcript data showed homoscedasticity and the null transcript data showed non-homoscedasticity. Thus, we used Bonferroni’s parametric test and the non-parametric Games-Howell test, respectively, for *post-hoc* analysis. All primer sequences and PCR product lengths are listed in Supplementary Tables S1 and S2.

### Protein extraction and WGA glycoprotein enrichment

Eyes were enucleated and retinas extracted in PBS and frozen. Subsequently, they were homogenized in lysis buffer (50 mM Tris pH 8.0, 100 mM NaCl, 1% Triton X-100) supplemented with EDTA-free Protease Inhibitor Cocktail (Roche Applied Science, Mannheim, Germany), containing 0.5 μg/ml leupeptin, 1 μg/ml pepstatin and 0.1 mM PMSF. Protein quantitation was made using the BCA Protein Assay Kit (Pierce, Rockford, IL, USA). For WGA enrichment, 350 μg of mouse retinal lysate was incubated with 100 μl of WGA-agarose beads (Vector Labs, Burlingame, CA, USA) in lectin-binding buffer (LBB: 20 mM Tris pH 7.5, 1 mM MnCl_2_, 1 mM CaCl_2_) at 4 °C overnight. Then, the beads were washed twice with LBB and boiled for 5 min in Laemmli sample buffer. The eluted proteins were used for western blotting and laminin-binding assays.

### Western blotting and laminin-binding assay

Proteins in WGA-enriched retinal extracts were separated by SDS-PAGE on 7.5% polyacrylamide gels and transferred to PVDF membranes (Immobilon-P, Millipore; Darmstadt, Germany). The membranes were blocked with 3% BSA in TBST (50 mM Tris, pH 7.5, 150 mM NaCl, 0.05% Tween-20) supplemented with 1 mM MnCl_2_ plus 1 mM CaCl_2_, and then incubated with the primary antibodies described in Supplementary Table S3, in the same blocking solution at 4 °C overnight. After washing with TBST, the membranes were incubated with the corresponding secondary antibodies (Supplementary Table S4) at a 1:5,000 dilution for 1 h, and the signal was detected using ECL reagents (Pierce). For posterior laminin-binding assays, antibodies were stripped by incubation of the membranes at 50 °C for 30 min in a buffer containing 100 mM β-mercaptoethanol, 2% SDS and 62.5 mM Tris, pH 6.7. Laminin-binding assays were performed as described previously^42^. Briefly, the membranes were incubated with 1.25 μg/ml laminin (L2020; Sigma-Aldrich, St. Louis, MO, USA) diluted in laminin-binding buffer (LamBB: 3% BSA in TBST supplemented with 1 mM CaCl_2_, 1 mM MgCl_2_ and 1 mM MnCl_2_) at 4 °C overnight. Then, they were blocked with LamBB for 1 h at room temperature, and thereafter sequentially incubated with an anti-laminin primary antibody and secondary antibodies, as above (Supplementary Tables S3 and S4). Signals were detected using ECL reagents.

### Electroretinography

Prior to ERG recording, mice were adapted to the dark overnight. Then, controls and *Pomt1* cKO mice were anesthetized under dim red light upon injection of ketamine (95 mg/kg, i.p.) and xylazine (5 mg/kg, i.p.) and maintained on a heated pad at 37 °C. Pupils were dilated by topical application of 1% tropicamide (Alcon Cusí, S.A., El Masnou, Barcelona, Spain) and flash-induced ERG responses were recorded from both eyes in response to light stimuli produced with a Ganzfeld stimulator. The setup procedures were conducted in dim red light for *ca*. 10 min, after which scotopic ERG (light flashes from −4 log cd·s·m^−2^ to 2 log cd·s·m^−2^) were recorded. Under scotopic conditions, five to ten responses were averaged; flash intervals were set at 30 s. After the dark-adapted ERG recordings, the animals were light-adapted for 10 min under white rod-suppressing background light (100 cd·m^−2^), and photopic ERG (light flashes from −0.8 log cd·s·m^−2^ to 2.1 log cd·s·m^−2^) were recorded. Under photopic conditions, 30 to 60 responses were averaged and flash intervals were set at 1 s. Calibration of the flash stimulating light source was performed using a Mavo-Monitor USB Light Meter (Gossen, Nürenberg, Germany).

The outcome measures were the response amplitudes and implicit times of each ERG component. The ERG complied with the International Society for Clinical Electrophysiology of Vision (ISCEV) standards^56^. The ERG signals were amplified and band filtered between 1 and 1000 Hz with a Grass CP511 AC amplifier (Grass Instruments, Quincy, MA, USA). Electrical signals were digitized at 20 kHz with a Power Laboratory data acquisition board (AD Instruments, Chalgrove, UK). Bipolar recordings were obtained using an Ag:AgCl mouse electrode fixed on a Burian-Allen corneal lens (Hansen Ophthalmic Development Lab, Coralville, IA, USA), a reference electrode located in the mouth, and a ground electrode located on the tail. Simultaneous recording was performed on both eyes. Each electrode was mounted on a coarse micromanipulator for easy positioning over the mouse eyes. Impedance of active and reference electrodes was less than 20 Ω.

### Other analyses

The optomotor response and visual water task were evaluated as described in Supplementary Materials and Methods, together with immunohistochemistry and electron microscopy assays.

## Electronic supplementary material

Supplementary Information
