# Supplementary material for: Impairment of photoreceptor ribbon synapses in a novel Pomt1 conditional knockout mouse model of dystroglycanopathy
Source: Sci Rep. 2018 Jun 4;8:8543. doi: 10.1038/s41598-018-26855-x (PMC5986861; doi:10.1038/s41598-018-26855-x)
Supplement: Supplementary file 1 — Supplementary Information [file 41598_2018_26855_MOESM1_ESM.pdf]

Supplementary Information

Impairment of photoreceptor ribbon synapses in a novel *Pomt1* conditional knockout mouse model of dystroglycanopathy

Marcos Rubio-Fernández[1,7], Mary Luz Uribe[2,7], Javier Vicente-Tejedor[3,7], Francisco Germain[3], Cristina Susín-Lara[1], Cristina Quereda[2], Lluis Montoliu[4,5], Pedro de la Villa[3,8], José Martín-Nieto[2,6,8], and Jesús Cruces[1,8]*

[1]Departamento de Bioquímica, Instituto de Investigaciones Biomédicas "Alberto Sols" UAM-CSIC, Facultad de Medicina, Universidad Autónoma de Madrid, 28029 Madrid, Spain.

[2]Departamento de Fisiología, Genética y Microbiología, Facultad de Ciencias, Universidad de Alicante, 03080 Alicante, Spain.

[3]Departamento de Biología de Sistemas, Facultad de Medicina, Universidad de Alcalá, 28805 Madrid, Spain.

[4]Departamento de Biología Molecular y Celular, Centro Nacional de Biotecnología, Consejo Superior de Investigaciones Científicas (CSIC), 28049 Madrid, Spain.

[5] Centro de Investigación Biomédica en Red de Enfermedades Raras (CIBERER), Instituto de Salud Carlos III, 28029 Madrid, Spain.

[6]Instituto Multidisciplinar para el Estudio del Medio "Ramón Margalef", Universidad de Alicante, 03080 Alicante, Spain.

[7] Co-first authors

[8] Co-senior authors

* Corresponding author

Name: Jesús Cruces.

Complete address: Lab. B-17, Departamento de Bioquímica, Facultad de Medicina, Universidad Autónoma de Madrid, 28029 Madrid, Spain.

Email: jesus.cruces@uam.es

Telephone number: +34 914975406.

Supplementary Figures

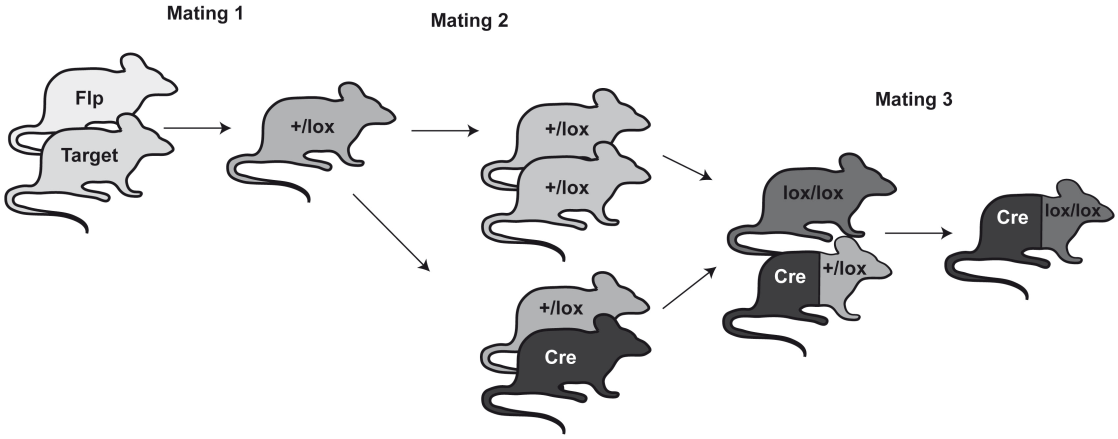

Supplementary Figure S1. Generation of *Pomt1* cKO mice. Mating schedule to generate *Pomt1* cKO mice. The different matings carried out and the genotypes used are shown. Flp, *flippase* transgenic mice; Cre, *Crx*-*Cre*$^{+}$ transgenic mice; Target, mice bearing the *Pomt1* targeted allele in heterozygosis; +/lox, mice bearing the *Pomt1* floxed allele in heterozygosis; lox/lox, mice bearing the *Pomt1* floxed allele in homozygosis; Cre +/lox, mice bearing both the *Pomt1* floxed allele in heterozygosis and the *Crx*-*Cre* transgene; Cre lox/lox, mice bearing both the *Pomt1* floxed allele in homozygosis and the *Crx*-*Cre* transgene.

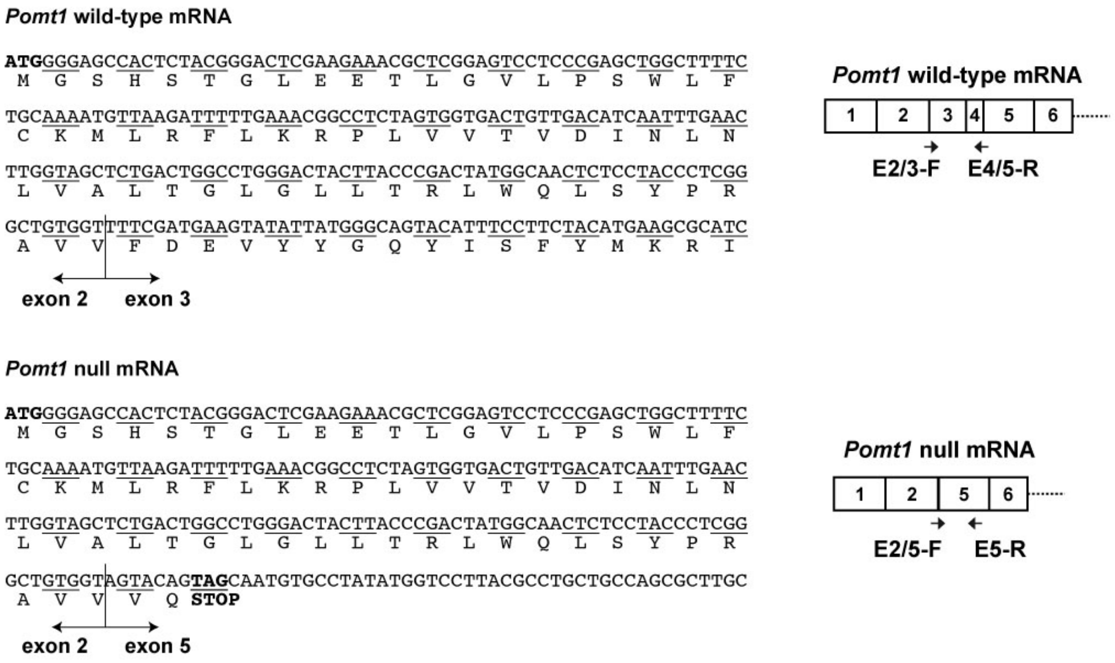

Supplementary Figure S2. Wild-type and null *Pomt1* mRNA sequences. Sequences of *Pomt1* mRNA wild-type and null mRNA transcripts. The ATG initiation codon and the TAG premature stop codon, the latter created by the removal of exons 3 and 4, are shown in bold in the *Pomt1* mRNA sequence. Exon borders are also depicted. On the right side, schemes are shown of the two expected transcripts and the annealing positions of primers used in RT-PCR and qRT-PCR assays.

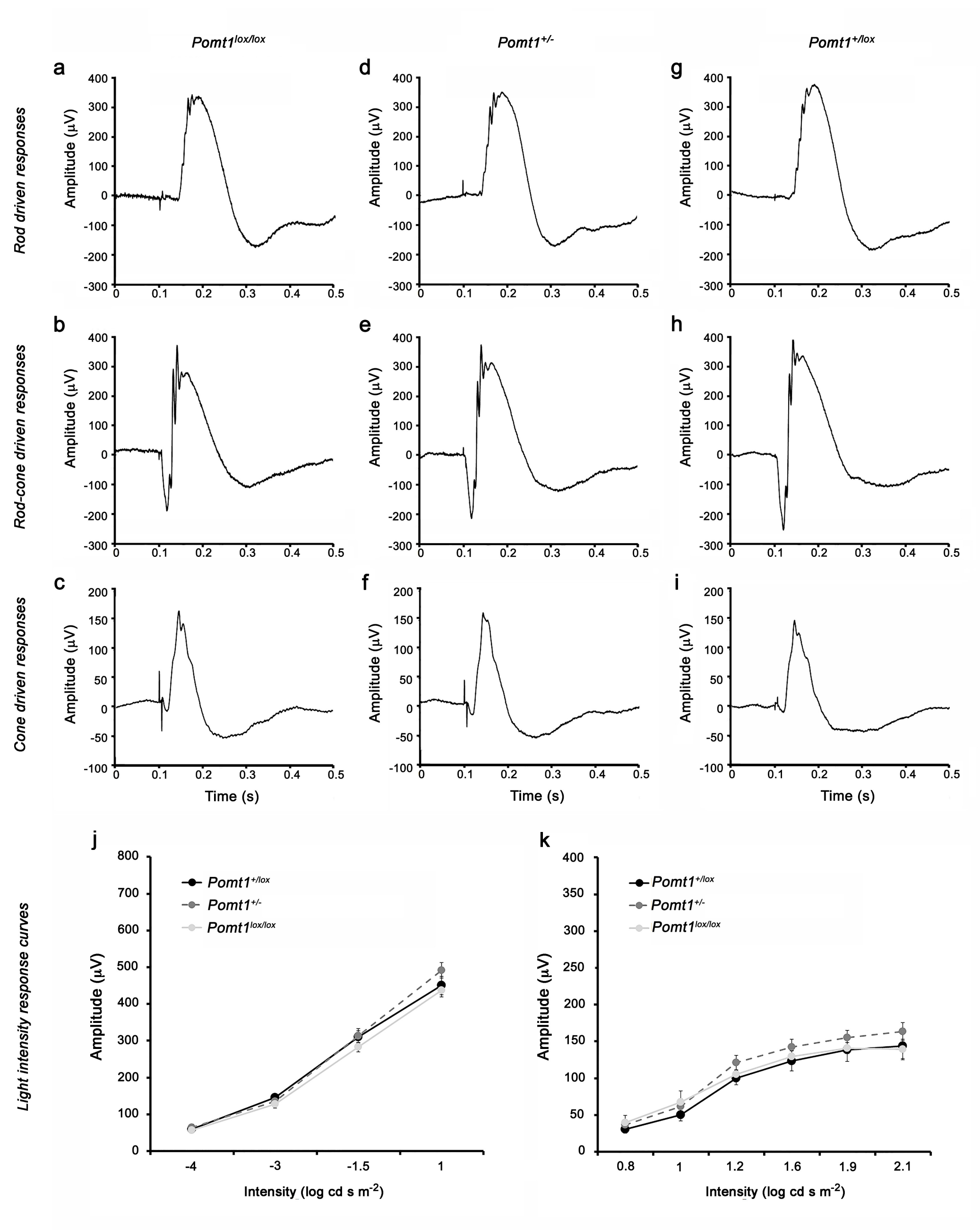

Supplementary Figure S3. Functional retinal responses in *Pomt1* genotypically-different control mice. ERG responses recorded from representative control mice with *Pomt1*$^{lox/lox}$ (**a–c**), *Pomt1*$^{+/–}$ (**d–f**) and *Pomt1*$^{+/lox}$ (**g–i**) genotypes are shown, recorded under scotopic (rod-driven responses; **a,d,g**), medium (rod plus cone-driven; **b,e,h**) and photopic (cone-driven; **c,f,i**) conditions. Light intensity-response curves obtained under scotopic (**j**) and medium (**k)** illuminance conditions are also shown for the three genotypes. Values are represented as the mean ± SEM (n = 6 mice per group). For light intensities, see the legend of **Figure 3**.

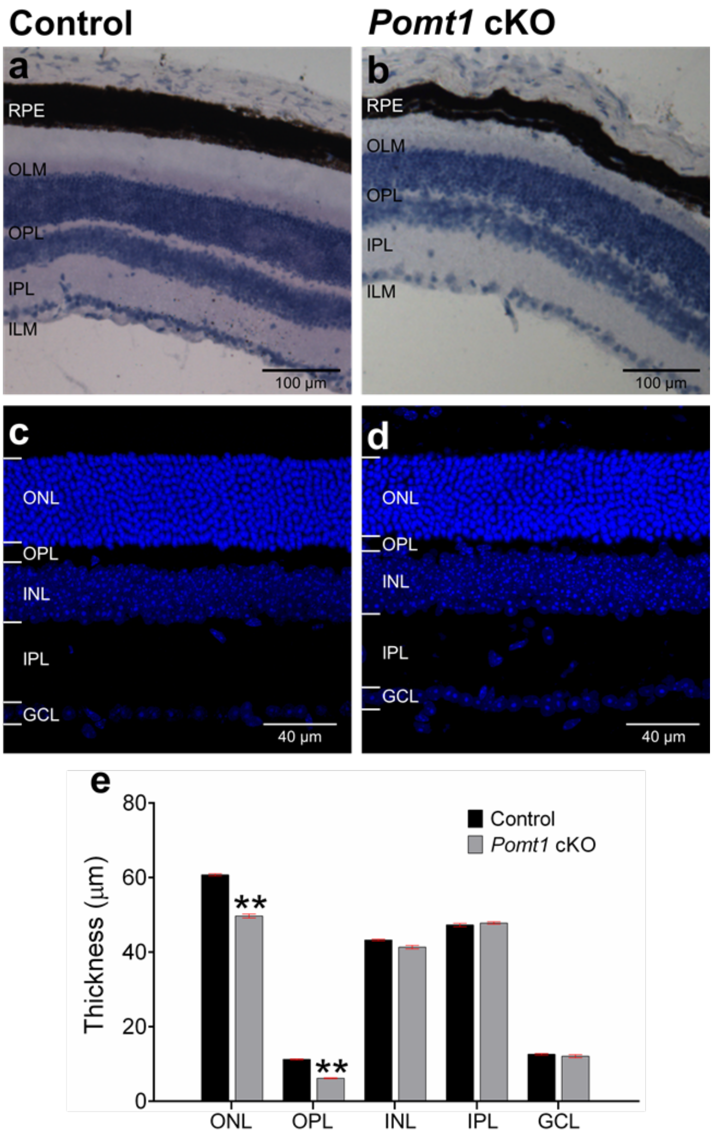

Supplementary Figure S4. Thickness of retinal layers in control and *Pomt1* cKO mice. Sections of control (*Pomt1*$^{+/lox}$) (**a**,**c**) and *Pomt1* cKO (**b**,**d**) retinas stained with hematoxylin and eosin (**a**,**b**) and the nuclear dye DAPI in blue (**c**,**d**). (**e**) Thickness of retinal layers in control and *Pomt1* cKO mice. No difference was observed between the thicknesses of the inner layers (INL, IPL and GCL), but significant thinning of the ONL and OPL was present in the *Pomt1* cKO as compared with control mice. Bars represent the mean ± SEM (n = 4 mice per group), **, $p < 0.001$. Abbreviations: RPE, retinal pigment epithelium; OLM, outer limiting membrane; OPL, outer plexiform layer; IPL, inner plexiform layer; ILM, inner limiting membrane; ONL, outer nuclear layer; INL, inner nuclear layer; GCL, ganglion cell layer.

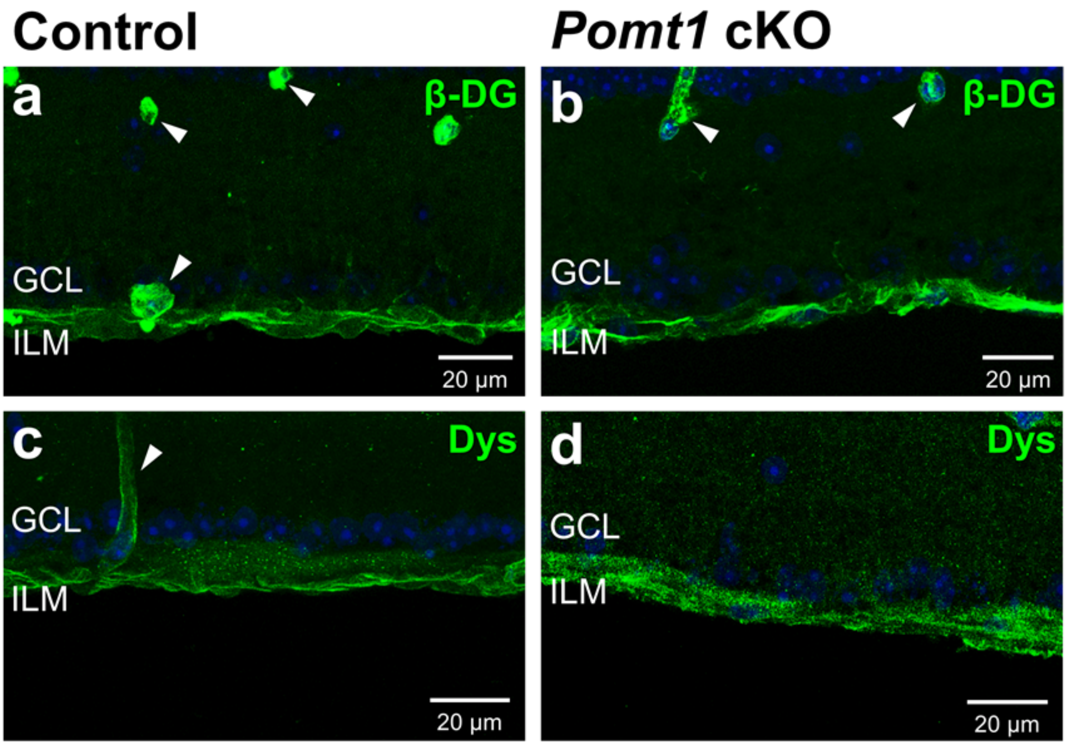

Supplementary Figure S5. Retinal inner limiting membrane in control and *Pomt1* cKO mice. Retinal sections from control (*Pomt1*$^{+/lox}$) (**a**,**c**) and *Pomt1* cKO (**b**,**d**) mice were stained with antibodies against β-dystroglycan (β-DG; **a**,**b**) or dystrophin (Dys; **c**,**d**). No significant differences in immunoreactivity along the ILM and in retinal blood vessels (arrowheads) were observed between the retinas of control and *Pomt1* cKO mice. All sections were co-stained with DAPI to show nuclei (blue). Abbreviations: GCL, ganglion cell layer; ILM, inner limiting membrane.

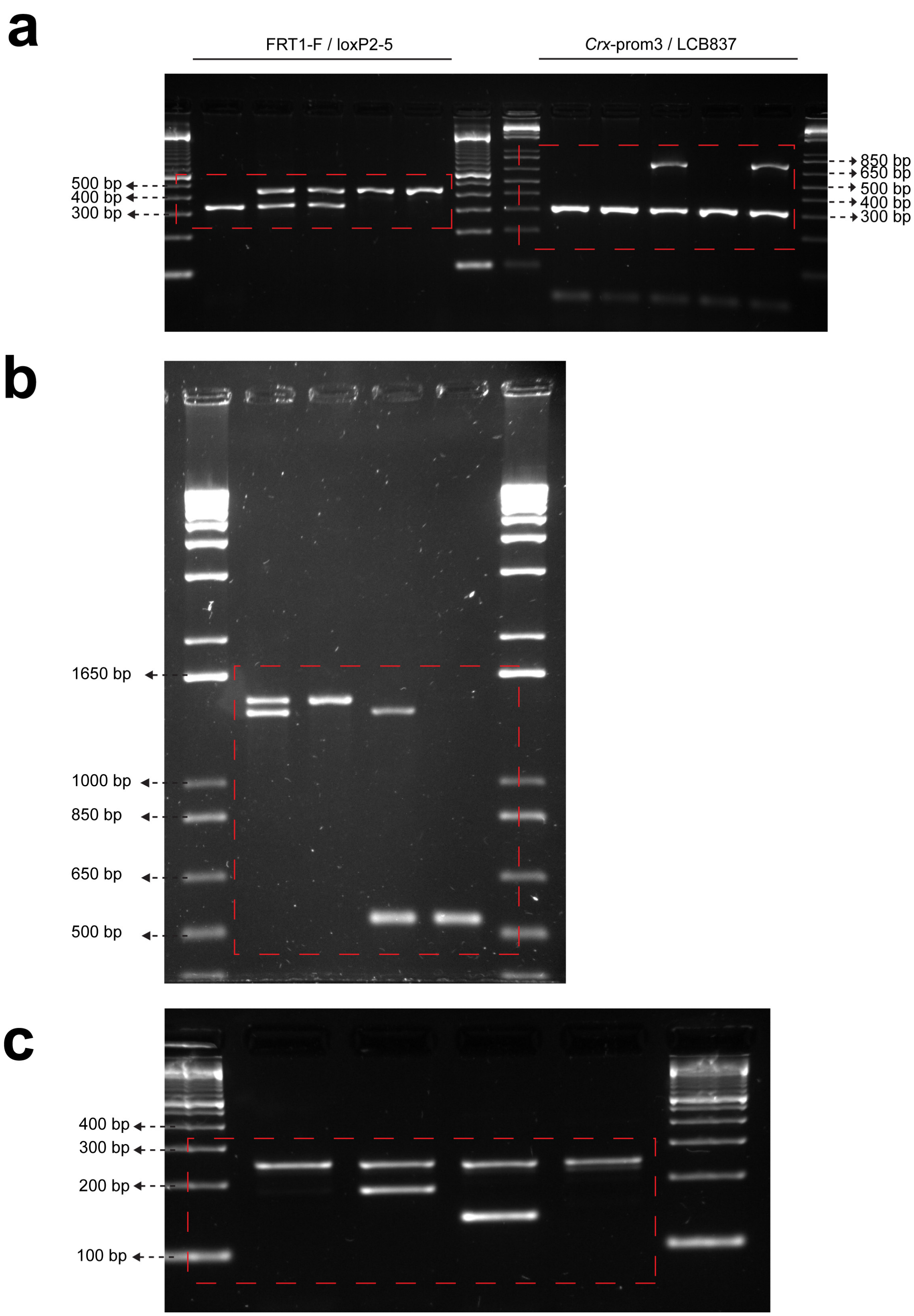

Supplementary Figure S6. Uncropped DNA electrophoresis gels. Images correspond to **Figure 1b** (**a**), **Figure 2a** (**b**) and **Figure 2b** (**c**). The red dashed line delineates the cropped area shown in each case.

a

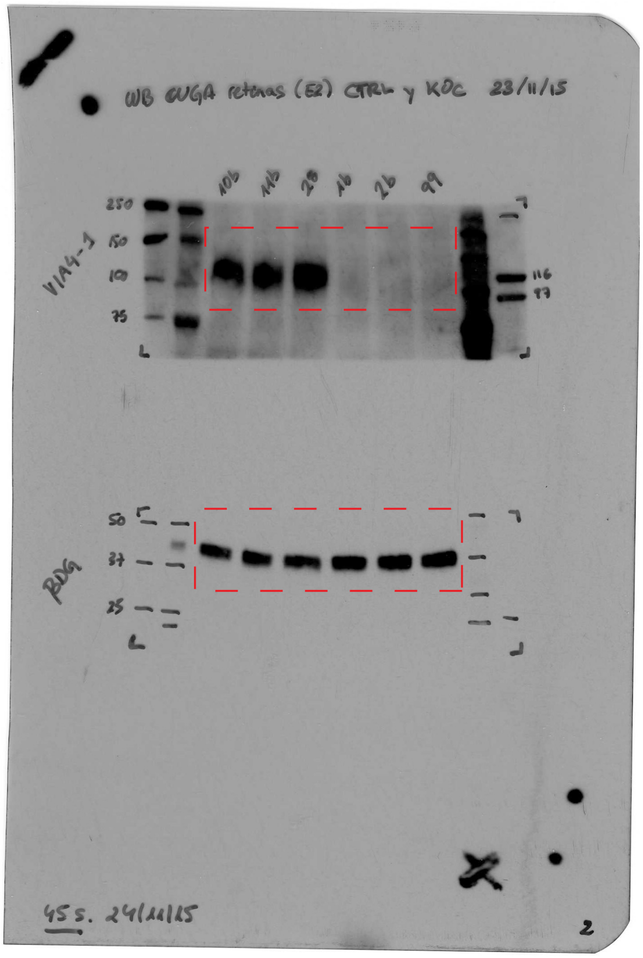

b

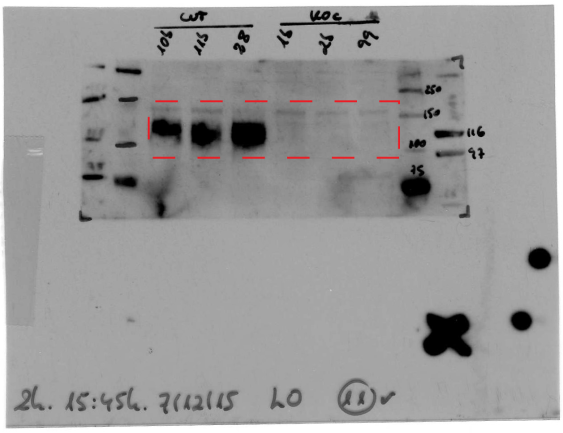

Supplementary Figure S7. Uncropped western blots and laminin overlay. Images correspond to VIA4-1 and β-DG western blots (**a**) and laminin overlay (**b**) presented in **Figure 2e**. The red dashed line delineates the cropped area shown in each case.

Supplementary Materials and Methods

Optomotor response. This behavioral assay was used to analyze reflexive optomotor responses. Mice reflexively respond to rotating vertical bars by characteristic movement of their head in the same direction and velocity of bars rotation. A homemade optomotor device was built based on the Prusky *et al.* design[1]. Mice were placed in the center of a square array of computer monitors that displayed stimulus gratings. We monitored the mouse using an overhead infrared television camera placed on top of the testing chamber. The test started with the easiest stimulus to mice, with a spatial frequency of 0.088 cycles/degree, a temporal frequency of 0.88 Hz and a normalized contrast of 1. Contrast sensitivity was calculated as the inverse of contrast threshold[2], and was measured at distinct spatial frequencies ranging from 0.011 to 0.355 cycles/degree. The Vision Egg tool was used for light stimulation. Stimuli consisted of vertical black/white bars (gratings) moving through the screens.

Water maze. The visual water task is used in behavioral experiments to study the visual acuity in animals and is based on the natural inclination of mice to escape from water. A homemade methacrylate trapezoidal-shaped tank with black walls was used. The front wall was transparent, allowing viewing of a light stimulus that was projected from a computer screen (monitor) located behind the frontal wall. Vision Egg software was used for stimulus programming. The stimulus consisted of vertical black/white bars (gratings) moving along the right-left screen sides. A midline divider was placed in the tank, which extended into the pool perpendicularly from the transparent wall, creating a maze with a stem and two arms. A transparent moveable platform was submerged in the pool at the end of one of the arms, just behind the light stimulus. Tap water was added to the tank to a depth 10 cm at 24°C. Mice needed to associate stimuli and platform location. In the training phase of the experiment, the best contrast and medium spatial and temporal frequency was displayed on one monitor (+ stimulus) and

uniform gray of the same luminance was displayed on the other (– stimulus). Mice were released 8 times from the end of the pool opposite the monitors, the platform being switched randomly to the left and right arms four times.

Hematoxylin and eosin staining and immunohistochemistry. Mice were euthanized with CO_2. Eyecups from *Pomt1* cKO and controls were enucleated, fixed in 4% paraformaldehyde in 0.1 M PBS, pH 7.4, and cryoprotected sequentially in 20, 30 and 40% sucrose. They were then embedded in optimal cutting temperature compound (Miles Inc., Elkhart, IN, USA) and frozen under liquid N_2 before 14–16 µm sections were obtained in a cryostat. Hematoxylin and eosin staining was carried out as described[3]. For immunohistochemistry, retinal sections were processed following well established procedures[4,5]. After blocking the sections with 5% BSA in phosphate buffer (PB) for 1 h, they were incubated overnight with primary antibodies in PB supplemented with 1% Triton X-100 (PBX) at room temperature. Then, primary antibodies were used at the dilutions specified in **Supplementary Table S3**, and thereafter for 1 h in the dark in PBX containing the corresponding Alexa Fluor®-conjugated secondary antibodies (**Supplementary Table S4)** at a 1:100 dilution. Fluorescein-conjugated peanut agglutinin (FL-1071, 1:100; Vector Labs) was used to label the base of cone pedicles, and DAPI (D1306, 10 µg/ml; Thermo Fisher Scientific) was simultaneously applied to stain the nuclei. Finally, the sections were coverslipped with Citifluor AF1 antifadent solution (Agar Scientific, London, UK), and fluorescence was detected with a Leica TCS SP2 confocal laser-scanning microscope. Immunohistochemical controls were performed by omitting the primary antibody. All immunostaining images shown are representative examples of three or more experiments, and were taken in the central area of the retina (near the optic nerve head) to ensure a valid comparison between animals. ImageJ software (v.1.51, NIH) was used for the morphometric measures and the data were analyzed using a two-tailed unpaired Student’s t-test with GraphPad v.6 Prism software.

Electron microscopy. Retinal tissue fixation and embedment for conventional electron microscopy was performed following previous protocols[6]. In brief, after separating the anterior pole of the eye, the eyecup was fixed by immersion in a 2% paraformaldehyde plus 2% glutaraldehyde solution in 0.1 M cacodylate buffer (CB), pH 7.4, for 2 h at room temperature with shaking. Then, retinas were washed in CB and post-fixed in 1% OsO_4, 1% potassium ferricyanide in H_2O for 2 h at 4ºC in the dark with shaking. Later, a graded series of ethanol was used for dehydration. Finally, retinas were exposed to propylene oxide and embedded in Epon 812 resin. Sectioning for electron microscopy examination was done with a Vitracut E ultramicrotome (Reichert-Jung, Vienna, Austria) in the transverse plane (1–2 μm). Ultra-thin sections (60–80 nm thick) were stained with lead citrate and viewed under a Zeiss M-10 transmission electron microscope (Carl Zeiss AG, Oberkochen, Germany).

Supplementary Tables

Table S1. Primers used for genotyping during generation of *Pomt1* cKO mice.

Gene	Primer ID	Sequence (5'→3')	PCR fragment size (bp)
lacZ	lacZ-F	CGTCGTTTTACAACGTCGTGAC	345
	lacZ-R	TAACAACCCGTCGGATTCTC	
Pomt1 loxP	FRT1-F	CATGCTCACTGGGCTTGGTAAAT	Wt: 328<br>Floxed: 431
	loxP2-R	CATGTGTGTAGTGTCCACCGAGG	
Crx-Cre	Crx-prom3	GCTGAAGCTGGAGGAATACCCTTTAGAC	797
	LCB837	AGCATTGCTGTCACTTGGTC	
IL2	oIMR0042	CTAGGCCACAGAATTGAAAGATCT	324
	oIMR0043	GTAGGTGGAAATTCTAGCATCATCC	
Pomt1	Pomt1-i2-F	ACATCCATGCAGGGGAGAAAAAGAC	Wt: 1,392<br>Floxed: 1,475<br>Null: 545
	Pomt1-i4-R	GTACCAAGAGCCAGGGATGGAATAG	

-F, forward; -R, reverse; Wt, wild-type allele; Floxed, floxed allele containing *loxP*; Null, null allele.

Table S2. Primers used for RT and qRT-PCR assays of mRNA expression from *Pomt1* alleles and control genes.

Primer ID	Sequence (5'→3')	PCR fragment size (bp)
E2/3-F	CGGGCTGTGGTTTTCGATGA	182
E4/5-R	TTGCTACTGTACTCTGCTCCAAT	
E2/5-F	CTCGGGCTGTGGTAGTACAGT	136
E5-R	CAATGGCAGCACCGTGGGAA	
Gapdh-F	CTTCACCACCATGGAGAAGGC	238
Gapdh-R	GGCATGGACTGTGGTCATGAG	
Actb-F	CTCTGGCTCCTAGCACCATGAAGA	200
Actb-R	GTAAAACGCAGCTCAGTAACAGTCCG	

-F, forward; -R, reverse. The underlined sequence corresponds to exon 2 (E2/3-F and E2/5-F primers) or exon 5 (E4/5-R primer) sequences.

Table S3. Primary antibodies used in this work.

Target	Use	Dilution	Type	Manufacturer	Cat. No.
α-DG (VIA4-1)	WB	1:200	Mouse mAb	Merck-Millipore	05-298
β-DG		1:10,000	Rabbit pAb	Santa Cruz Biotech.	sc-28535
β-DG	IHC	1:100	Mouse mAb	Abcam	ab49515
Bassoon		1:1,000	Mouse mAb	Enzo Life Science	SAP7F407
Calbindin		1:500	Mouse mAb	Sigma-Aldrich	C94848
Cone arrestin		1:500	Rabbit pAb	Merck-Millipore	AB15282
CtBP2		1:1,000	Mouse mAb	BD Biosciences	612044
Dystrophin		1:100	Rabbit pAb	Abcam	ab15277
Laminin		1:1,000	Rabbit pAb	Sigma-Aldrich	L9393
Pikachurin		1:50	Rabbit pAb	ProteinTech Group	14578-1-AP
mGluR6		1:100	Rabbit pAb	Acris	RA13105
PKC-α		1:100	Rabbit pAb	Santa Cruz Biotech.	sc-10800
POMT1		1:100	Rabbit pAb	Thermo Fisher	PA5-66756
Rhodopsin		1:250	Mouse mAb	Merck-Millipore	MAB5356
Synaptophysin		1:500	Rabbit mAb	Abcam	ab52636

mAb, monoclonal antibody; pAb, polyclonal antibody; WB, western blotting; IHC, immunohistochemistry.

Table S4. Secondary antibodies used in this work.

Antibody	Use	Conjugate	Manufacturer	Cat. No.
Goat anti-mouse	WB	HRPO	Santa Cruz Biotech.	sc-2005
Goat anti-rabbit		HRPO	Santa Cruz Biotech.	sc-2004
Donkey anti-mouse	IHC	AF-488 (green)	Molecular Probes	A-21202
Donkey anti-rabbit		AF-488 (green)	Molecular Probes	A-21206
Donkey anti-rabbit		AF-546 (red)	Molecular Probes	A-10040
Donkey anti-mouse		AF-555 (red)	Molecular Probes	A-31570
Donkey anti-rabbit		AF-647 (far red)	Molecular Probes	A-31573

All antibodies were polyclonal and directed to IgG from the indicated species. WB, western blotting; IHC, immunohistochemistry; HRPO, horseradish peroxidase; AF, Alexa Fluor.

Supplementary References

1. Prusky, G. T., Harker, K. T., Douglas, R. M. & Whishaw, I. Q. Variation in visual acuity within pigmented, and between pigmented and albino rat strains. *Behav. Brain Res.* **136,** 339–348 (2002).
2. Umino, Y., Solessio, E. & Barlow, R. B. Speed, spatial, and temporal tuning of rod and cone vision in mouse. *J. Neurosci.* **28,** 189–198 (2008).
3. Fischer, A. H., Jacobson, K. A., Rose, J. & Zeller, R. Hematoxylin and eosin staining of tissue and cell sections. *CSH Protoc.* **3,** 4986–4988 (2008).
4. Esteve-Rudd, J., Campello, L., Herrero, M. T., Cuenca, N. & Martín-Nieto, J. Expression in the mammalian retina of parkin and UCH-L1, two components of the ubiquitin-proteasome system. *Brain Res.* **1352,** 70–82 (2010).
5. Uribe, M. L. *et al.* Expression pattern in retinal photoreceptors of POMGnT1, a protein involved in muscle-eye-brain disease. *Mol. Vis.* **22,** 658–673 (2016).
6. Jimeno, D. *et al.* RasGRF2 controls nuclear migration in postnatal retinal cone photoreceptors. *J. Cell Sci.* **129,** 729–742 (2016).
